# A quantitative model using multi-parameters in dual-energy CT to preoperatively predict serosal invasion in locally advanced gastric cancer

**DOI:** 10.1186/s13244-024-01844-z

**Published:** 2024-10-31

**Authors:** Yiyang Liu, Mengchen Yuan, Zihao Zhao, Shuai Zhao, Xuejun Chen, Yang Fu, Mengwei Shi, Diansen Chen, Zongbin Hou, Yongqiang Zhang, Juan Du, Yinshi Zheng, Luhao Liu, Yiming Li, Beijun Gao, Qingyu Ji, Jing Li, Jianbo Gao

**Affiliations:** 1 Department of Radiology, The First Affiliated Hospital of Zhengzhou University, Zhengzhou, 450052 China; 2 Henan International Joint Laboratory of Medical Imaging, Zhengzhou, China; 3 Henan Key Laboratory of Image Diagnosis and Treatment for Digestive System Tumor, Zhengzhou, China; 4 Henan Key Laboratory of CT Imaging, Zhengzhou, China; 5 Department of Radiology, The Affiliated Cancer Hospital of Zhengzhou University (Henan Cancer Hospital), Zhengzhou, 450008 China; 6 Department of Gastrointestinal Surgery, The First Affiliated Hospital of Zhengzhou, University, Zhengzhou, 450052 China; 7 Department of Radiology, The Second Affiliated Hospital of Baotou Medical College, Inner Mongolia University of Science and Technology, Baotou, 014030 China; 8 Department of Radiology, The First Affiliated Hospital of Henan University of Science and Technology, Luoyang, 471003 China; 9 CT Diagnostic Center, Sanmenxia Central Hospital, Sanmenxia, 472000 China; 10 Medical Imaging Center, The First People’s Hospital of Shangqiu City, Shangqiu, 476100 China; 11 College of Acupuncture and Massage, Henan University of Chinese Medicine, Zhengzhou, 450046 China

**Keywords:** X-ray computed, Dual-energy CT, Multi-parameters, Serosal invasion

## Abstract

**Objectives:**

To develop and validate a quantitative model for predicting serosal invasion based on multi-parameters in preoperative dual-energy CT (DECT).

**Materials and methods:**

A total of 342 LAGC patients who underwent gastrectomy and DECT from six centers were divided into one training cohort (TC), and two validation cohorts (VCs). Dual-phase enhanced DECT-derived iodine concentration (IC), water concentration, and monochromatic attenuation of lesions, along with clinical information, were measured and collected. The independent predictors among these characteristics for serosal invasion were screened with Spearman correlation analysis and logistic regression (LR) analysis. A quantitative model was developed based on LR classifier with fivefold cross-validation for predicting the serosal invasion in LAGC. We comprehensively tested the model and investigated its value in survival analysis.

**Results:**

A quantitative model was established using IC, 70 keV, 100 keV monochromatic attenuations in the venous phase, and CT-reported T4a, which were independent predictors of serosal invasion. The proposed model had the area-under-the-curve (AUC) values of 0.889 for TC and 0.860 and 0.837 for VCs. Subgroup analysis showed that the model could well discriminate T3 from T4a groups, and T2 from T4a groups in all cohorts (all *p* < 0.001). Besides, disease-free survival (DFS) (TC, *p* = 0.015; and VC1, *p* = 0.043) could be stratified using this quantitative model.

**Conclusion:**

The proposed quantitative model using multi-parameters in DECT accurately predicts serosal invasion for LAGC and showed a significant correlation with the DFS of patients.

**Critical relevance statement:**

This quantitative model from dual-energy CT is a useful tool for predicting the serosal invasion of locally advanced gastric cancer.

**Key Points:**

- Serosal invasion is a poor prognostic factor in locally advanced gastric cancer that may be predicted by DECT.
- DECT quantitative model for predicting serosal invasion was significantly and positively correlated with pathologic T stages.
- This quantitative model was associated with patient postoperative disease-free survival.

**Graphical Abstract:**

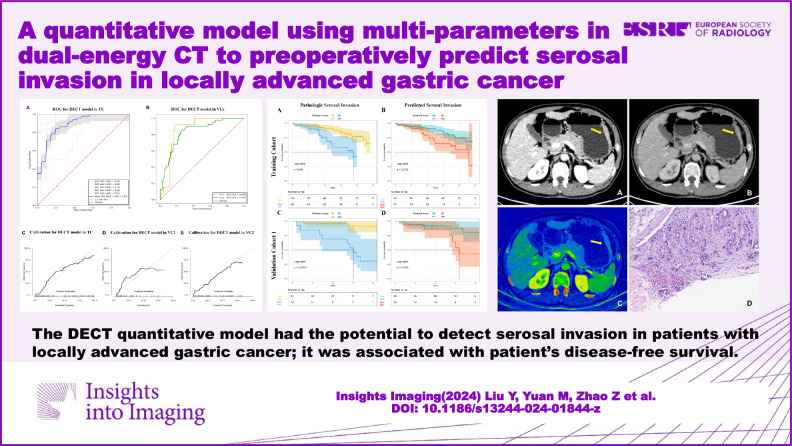

## Introduction

Gastric cancer (GC) remains a highly lethal malignancy worldwide and is responsible for 659,853 deaths in 2022 [1]. Metastatic progression is the ultimate cause of death in GC patients [2]. Peritoneal metastasis accounts for 53% to 66% of cases in patients with distant metastatic GC, establishing it as the predominant route of metastasis [3, 4]. Patients with serosal invasion (SI) at the baseline period are at high risk for micro-metastases and peritoneal spreading [5]. Moreover, studies have shown that SI is an independent risk factor for GC recurrence after radical resection [6, 7]. However, the gold standard for diagnosing SI is postoperative histopathologic examination [8]. Hence, noninvasive preoperative knowledge of serosal status at the baseline is desirable and may assist surgeons in identifying high-risk populations and predicting patient’s prognoses.

Computed tomography (CT) imaging has been routinely used for preoperative T staging. On CT, an irregular or nodular outer margin of the serosal/outer layer, along with dense band-like fat infiltration around the stomach, indicates SI [9]. However, T2 and T3 tumors may be over-staged as T4 because perigastric infiltration from GC is difficult to distinguish from perigastric inflammation or fibrosis on CT [10]. Furthermore, conventional CT lacks functional parameters and thus evaluation of lesions is more by morphology, which may be a key reason for limiting the clinical usefulness of CT for GC.

Dual-energy CT (DECT), a novel CT technology, can acquire datasets containing both high and low-energy spectra in one scan. These two datasets, when input into a post-processing workstation, allow for the generation of rich clinical image sets, such as multiple virtual monochromatic images (VMIs) and iodine- and water-image pairs [11]. Iodine concentration (IC) obtained from DECT-derived iodine map (IM) has been proven to be strongly correlated with tumor angiogenesis [12]. It has also demonstrated encouraging diagnostic accuracy in determining metastatic lymph nodes, diagnosing perineural invasion, and decoding microsatellite instability status, as well as evaluating therapeutic efficacy for GC [13–16]. Additionally, previous studies have developed DECT logistic regression (LR) models incorporating iodine parameters that successfully predicted lymph node metastasis (LNM) and human epidermal growth factor receptor 2 (HER-2) status in GC [17, 18]. In assessing the depth of tumor infiltration, two studies have found that IC shows a significant difference between serosal invasion and non-serosal invasion [19, 20].

Therefore, the aim of this multicenter study was to investigate whether preoperative DECT parameters originated from the VMI sets and iodine-based material decomposition image, combined with conventional clinical features, could enable the accurate prediction of SI in LAGC and further evaluate the potential prognostic value of using these factors.

## Materials and methods

### Patients

**Fig. 1:**
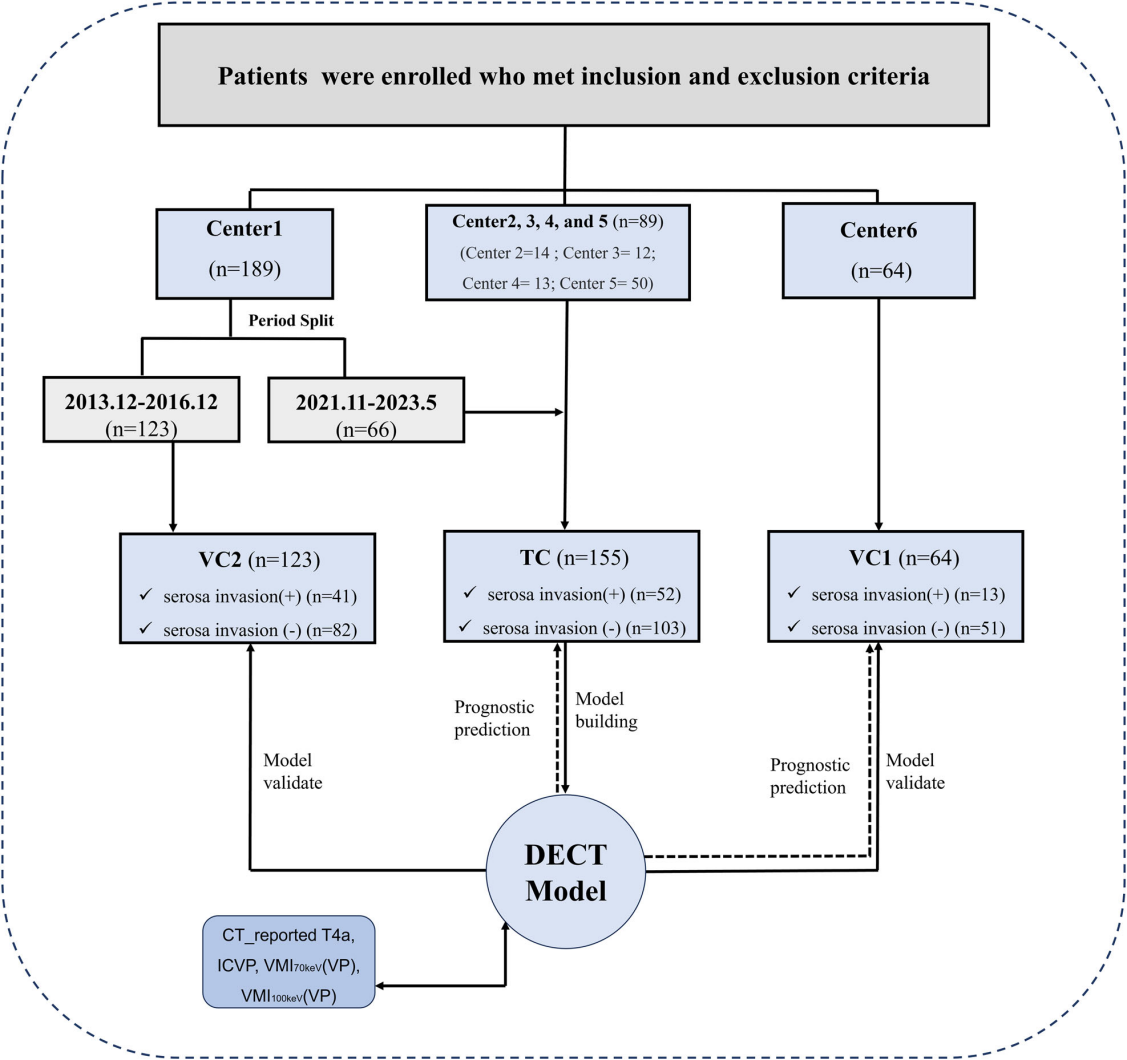
Flow diagram of the present study. DECT, dual-energy CT; IC, iodine concentration; VP, venous phase; VMI_70keV_, CT attenuation on 70-keV virtual monochromatic images; VMI_100keV_, CT attenuation on 100-keV virtual monochromatic images; TC, training cohort; VC, validation cohort

The institutional review board of all participating hospitals approved this multicenter study. This observational study encompassed prospective development and validation of a DECT model, along with retrospective validation, to assess reliability and generalizability. Of these, the prospective work has been registered (Registration number, ChiCTR2400082707). To develop the DECT model, patients with LAGC were prospectively recruited from Centers 1 through 5, comprising the training cohort (TC). External validation was performed using an independent cohort (validation cohort 1, VC1) prospectively enrolled from Center 6. For the retrospective validation, eligible patients were consecutively recruited from the clinical database of Center 1 (validation cohort 2, VC2). The main inclusion criteria were as follows: (1) underwent radical gastrectomy; (2) postoperative pathologically diagnosed as locally advanced gastric adenocarcinoma (pT2-4aNxM0); (3) dual-phase enhanced DECT scans under spectral imaging mode carried out less than 2 weeks before surgery; (4) available clinical and pathological data. The main exclusion criteria for patients enrolled were as follows: (1) poor DECT images quality; (2) patient underwent preoperative anti-cancer treatment; (3) difficult to measure the tumor on CT images because of insufficient stomach distension or the tumor was too small. Patient recruitment periods are listed in Supplementary Appendix S1. The flowchart of the overall study design is shown in Fig. 1.

The data on clinicopathologic characteristics were collected through electronic medical records. Pathologic evaluation for TNM stages was based on the Eighth edition of the American Joint Committee on Cancer (AJCC) tumor, node, metastasis (TNM) staging system [21].

### CT technique

CT examinations were acquired on DECT scanners equipped with the rapid tube voltage (kVp)-switching (GE HealthCare). For details on imaging protocols and patient preparation before examination, see Supplementary Table S1 and Appendix S2. The axial 40/70/100/140 keV VMIs, IM, and water maps were generated from the DECT datasets in the arterial phase (AP) and venous phase (VP).

### DECT image analysis

All DECT images were transferred to a commercially available workstation (AW4.7; GE HealthCare). The measurement and evaluation details are shown in Supplementary Appendix S3. Finally, we obtained seven DECT quantitative parameters from each phase scan. The mean value of each parameter was applied for statistical analysis.

### Model construction

A procedure for selecting independent predictors for SI was devised in the TC as follows: (1) Spearman correlation analysis, (2) univariable analysis, and (3) multivariate logistic regression. The details of feature selection are shown in Supplementary Appendix S4. Subsequently, a quantitative model was constructed by using the relevant predictors and the regression coefficients. The performance of the model was validated in the two independent cohorts.

### Model performance evaluation

The goodness of fit of the model was evaluated with a calibration curve accompanied by Hosmer-Lemeshow test. Moreover, stratification analysis based on clinicopathological characteristics was performed on the integrated dataset of the two validation cohorts. We further conducted subgroup analysis with Mann–Whitney *U*-test analysis and spearman rank correlation test for discriminating T2 versus T3, T2 versus T4, and T3 versus T4. In addition, we evaluated the associations between the model and N stage using Mann–Whitney *U*-test analysis.

### Follow-up

After surgery, patients received follow-up examinations using contrast-enhanced CT, MRI, or PET exams and/or endoscopy at 3–6-month intervals. The local recurrence or metastasis was identified by means of typical imaging features and/or pathological findings. Disease-free survival (DFS) is defined as the time (in months) from the date of surgery to the date of first disease local recurrence, progression, or death, while overall survival (OS) is defined as the time to death. The deadline date for the follow-up period was December 31, 2023. The median of follow-up duration was 15.4 months (range, 3.9–25.4 months).

## Statistical analysis

Differences in variables between the SI-positive and SI-negative groups were analyzed using the independent *t*-test or the Wilcoxon rank-sum test for continuous variables and the Fisher’s exact test or chi-squared for categorical variables. To obtain a robust model, the DECT model based on logistic regression classifier was trained with a fivefold cross-validation strategy in the TC. Area under the curve (AUC) of the receiver operating characteristic (ROC) curve was used to assess the discriminative ability of the developed model and each independent predictor. The optimal cutoff value was determined with the Youden index. Hanley–McNeil test was used to compare AUC value: (1) the DECT model among different cohorts; (2) the DECT model in stratification analysis. While the performance within and between individual predictors and DECT model was compared using the DeLong test. Survival analysis was performed for patients in the TC and VC1. Kaplan–Meier curves, the log-rank test, and univariate cox regression were applied to assess the prognostic significance of the pathologic SI and the DECT model.

Statistical analysis was performed using Python (version 3.8), and R software (version 4.3.2).

## Results

### Characteristics of the study sample

Of the 429 patients considered, 62 were excluded because they had received anti-cancer therapy, 14 because they had inadequate DECT images quality, and 11 because their tumors could not be measured.

A total of 342 GC patients were ultimately enrolled, with 155 patients (45.3%) in TC (mean age, 63 years ± 10 (SD); 124 men (80%) and 31 women (20%)), 64 (18.7%) in VC1 (mean age, 63 years ± 9; 51 men (79.7%) and 13 women (20.3%)), and 123 (36.0%) in VC2 (mean age, 59 years ± 11; 100 men (81.3%) and 23 women (18.7%)). Clinicopathological data for patients in all three cohorts are presented in Supplementary Table S2. No significant differences were observed in sex, location, size (thickness), lymph node status, or differentiation degree between TC and two VCs (*p* > 0.05), except for patients’ age (*p* = 0.003).

### Prediction model construction

**Fig. 2:**
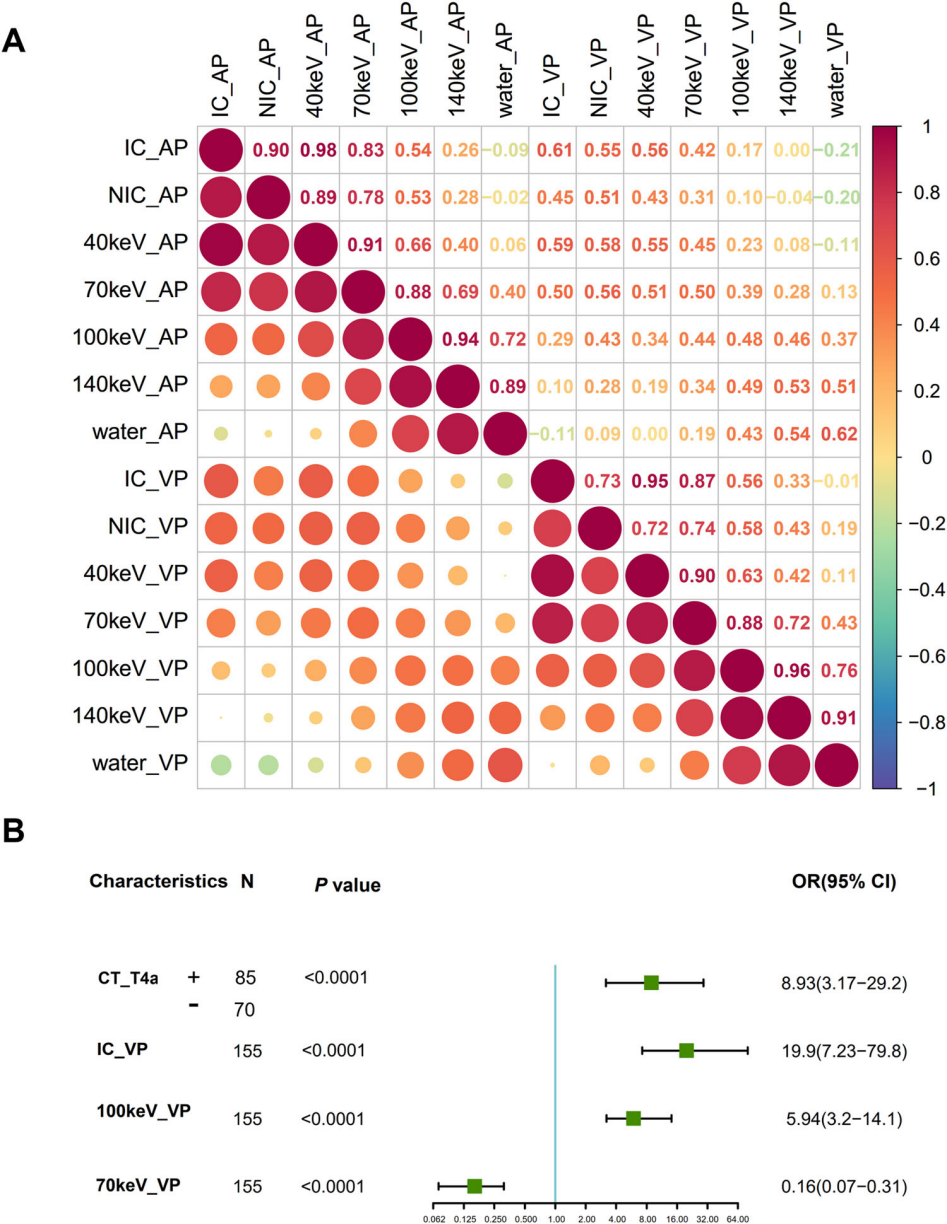
Heat map and forest plot. **A** Spearman correlation analysis of DECT parameters. **B** Forest plot illustrating characteristics identified as significant via multivariable logistic regression for the training cohort (*n* = 155). Odds ratios (ORs) are presented, with 95% CIs in parentheses. IC, iodine concentration; nIC, normalized iodine concentration; AP, arterial phase; VP, venous phase; WC, water concentration; 40/70/100/140 keV, CT attenuation on 40/70/100/140-keV virtual monochromatic images

Correlation analysis in the TC showed that the VMI_40keV_ attenuation (AP), VMI_140keV_ attenuation (AP), VMI_40keV_ attenuation (VP), and VMI_140keV_ attenuation (VP) had significant linear correlations with other DECT parameters and were therefore eliminated (Fig. 2A). After that, among the remaining non-redundant features in the TC, IC (VP), VMI_70keV_ attenuation (VP), VMI_100keV_ attenuation (VP), WC (VP), Location, and CT-reported T4a (all *p* < 0.05) were significantly associated with the pathologic SI (Table 1). Then these significant, non-redundant variables were included in stepwise multivariable logistic analysis, and finally, IC (VP), VMI_70keV_ attenuation (VP), VMI_100keV_ attenuation (VP), and CT-reported T4a were identified as independent predictors for serosal invasion (all *p* < 0.001), with OR (95% CI) values of 19.950 (7.23, 79.8), 0.165 (0.07, 0.31), 5.943 (3.20, 14.1), and 8.935 (3.17, 29.2), respectively (Table 2). These predictors are presented as a forest plot to show the weight of individual characteristics (Fig. 2B). Subsequently, a hybrid DECT model that integrated corresponding independent predictors was developed. By applying the linear predictors based on the regression coefficients and the logistic function (sigmoid function), the estimated probability of SI could be calculated as follows: Probability = 1/[1+exp (4.102 − 0.554 × CT-reported T4a (positive) − 0.861 × IC (VP) + 0.486 × VMI_70keV_(VP) − 0.493 × VMI_100keV_(VP))].

**Table 1:** Comparison of clinicopathological characteristics and DECT parameters between the serosal invasion-positive and serosal invasion-negative groups in the training and validation cohorts

Variables	Training cohort			Validation cohort 1			Validation cohort 2		
	Non-serosal invasion (*n* = 103)	Serosal invasion (*n* = 52)	*p*-value	Non-serosal invasion (*n* = 51)	Serosal invasion (*n* = 13)	*p*-value	Non-serosal invasion (*n* = 82)	Serosal invasion (*n* = 41)	*p*-value
Sex			0.269			1.000			0.744
Male	85 (82.5%)	39 (75.0%)		41 (80.4%)	10 (76.9%)		16 (19.5%)	7 (17.1%)	
Female	18 (17.5%)	13 (25.0%)		10 (19.6%)	3 (23.1%)		66 (80.5%)	34 (82.9%)	
Age (years)	61.961 ± 9.970	64.788 ± 9.375	0.091	63.588 ± 8.372	61.077 ± 9.106	0.346	59.329 ± 10.625	58.707 ± 11.695	0.768
Location			0.034*			0.154			0.329
Upper	45 (43.7%)	13 (25.0%)		26 (51.0%)	5 (38.5%)		33 (40.2%)	11 (26.8%)	
Middle	13 (12.6%)	12 (23.1%)		9 (17.6%)	0 (0.0%)		10 (12.2%)	9 (22.0%)	
Lower	33 (32.0%)	24 (46.2%)		9 (17.6%)	4 (30.8%)		32 (39.0%)	16 (39.0%)	
Diffuse^b^	12 (11.7%)	3 (5.8%)		7 (13.7%)	4 (30.8%)		7 (8.5%)	5 (12.2%)	
Thickness (mm)	17.650 ± 7.024	17.102 ± 6.451	0.638	14.600 (11.625–17.950)	16.200 (13.870–17.300)	0.301	16.354 ± 6.744	16.775 ± 6.438	0.741
CT-reported T4a			< 0.001**			0.003**			0.610
Negative	59 (57.3%)	11 (21.2%)		35 (68.6%)	3 (23.1%)		44 (53.7%)	20 (48.8%)	
Positive	44 (42.7%)	41 (78.8%)		16 (31.4%)	10 (76.9%)		38 (46.3%)	21 (51.2%)	
CT-reported_LNM			0.396			0.560			0.251
Negative	49 (47.6%)	21 (40.4%)		13 (25.5%)	5 (38.5%)		43 (52.4%)	17 (41.5%)	
Positive	54 (52.4%)	31 (59.6%)		38 (74.5%)	8 (61.5%)		39 (47.6%)	24 (58.5%)	
Differentiation degree			0.176			0.092			0.071
Non-poorly differentiated	67 (65.0%)	28 (53.8%)		25 (49.0%)	3 (23.1%)		52 (63.4%)	19 (46.3%)	
Poorly differentiated	36 (35.0%)	24 (46.2%)		26 (51.0%)	10 (76.9%)		30 (36.6%)	22 (53.7%)	
IC_AP (100 µg/mL)	17.400 ± 7.450	18.027 ± 8.671	0.64	12.720 (9.680–17.565)^a^	10.560 (8.500–13.180)^a^	0.099	10.945 (8.373–14.322)^a^	16.190 (11.520–20.720)	0.001**
NIC_AP	0.145 ± 0.063	0.135 ± 0.065	0.367	0.161 (0.107–0.247)^a^	0.119 (0.096–0.147)^a^	0.055	0.144 ± 0.076	0.183 ± 0.095	0.016*
40keV_AP (HU)	172.729 ± 59.974	179.509 ± 71.235	0.534	140.710 (113.100–171.085)^a^	106.970 (102.200–135.100)^a^	0.109	115.495 (95.438–154.195)^a^	147.860 (113.450–191.350)	0.001**
70keV_AP (HU)	74.080 ± 20.227	76.937 ± 23.569	0.434	61.460 (51.675–72.230)^a^	59.860 (53.500–64.150)^a^	0.317	54.405 (46.485–68.775)^a^	69.510 (56.600–81.660)	0.000**
100keV_AP (HU)	49.097 ± 12.876	50.285 ± 12.446	0.584	44.260 ± 12.468	42.715 ± 7.381	0.671	42.439 ± 15.120	47.314 ± 10.532	0.067
140keV_AP (HU)	38.708 ± 11.640	39.385 ± 9.092	0.714	35.734 ± 10.898	35.977 ± 8.420	0.941	34.806 ± 12.183	36.745 ± 9.137	0.37
WC_AP (mg/cm^3^)	1029.540 (1022.265–1035.090)^a^	1029.440 (1026.197–1033.480)^a^	0.726	1028.158 ± 10.340	1030.028 ± 9.902	0.560	1027.924 ± 11.008	1028.570 ± 13.272	0.775
IC_VP (100 µg/mL)	21.036 ± 6.215	24.718 ± 7.866	0.002**	18.547 ± 6.908	19.490 ± 5.066	0.647	18.085 (15.117–22.902)^a^	23.770 (18.900–28.420)	0.000**
NIC_VP	0.460 ± 0.122	0.500 ± 0.141	0.071	0.444 (0.326–0.504)^a^	0.372 (0.320–0.437)^a^	0.133	0.433 ± 0.156	0.479 ± 0.152	0.127
40keV_VP (HU)	211.058 ± 59.362	232.983 ± 68.701	0.041*	188.803 ± 58.077	181.581 ± 39.773	0.674	180.270 (149.640–213.500)^a^	227.740 (179.160–264.610)	0.001**
70keV_VP (HU)	85.769 ± 18.816	95.789 ± 22.662	0.004**	79.605 ± 19.564	77.021 ± 13.237	0.655	76.116 ± 18.220	88.411 ± 21.309	0.001**
100keV_VP (HU)	54.156 ± 11.528	60.237 ± 12.022	0.003**	51.339 ± 11.122	49.738 ± 8.461	0.631	49.059 ± 11.516	55.518 ± 11.945	0.004**
140keV_VP (HU)	41.152 ± 10.404	45.696 ± 8.899	0.008**	39.762 ± 9.060	38.634 ± 7.797	0.682	37.364 ± 10.104	41.192 ± 10.181	0.05
WC_VP (mg/cm^3^)	1029.170 (1024.435–1035.555)^a^	1031.470 (1028.180–1036.062)^a^	0.049*	1029.278 ± 8.694	1028.696 ± 8.118	0.828	1027.167 ± 9.960	1028.403 ± 10.557	0.526

Values are displayed as the number (percentage) or mean value ± SD or median (interquartile range)

*AP* arterial phase, *IC* iodine concentration, *nIC* normalized iodine concentration, *VP* venous phase, *WC* water concentration, *40/70/100/140 keV* CT attenuation on 40/70/100/140-keV virtual monochromatic images

^a^ Variables are non-normally distributed

^b^ Diffuse ≥ 2/3 stomach

*p < 0.05, **p < 0.01

**Table 2:** Independent predictors of serosal invasion identified by stepwise multivariate logistic regression analysis

Variables	DECT model			
	β	Standard error	OR (95% CI)	*p*-value
CT-reported T4a (Positive)	2.190	0.561	8.935 (3.17, 29.2)	< 0.001
IC_VP	2.988	0.600	19.95 (7.23, 79.8)	< 0.001
VMI_70keV__VP	−1.8032	0.378	0.165 (0.07, 0.31)	< 0.001
VMI_100keV__VP	1.782	0.371	5.943 (3.20, 14.1)	< 0.001

These analyses were performed using the training dataset (*n* = 155)

*AP* arterial phase, *CI* confidence interval, *IC* iodine concentration, *OR* odds ratio, *VP* venous phase, *VMI*_*70keV*_ CT attenuation on 70-keV virtual monochromatic images, *VMI*_*100keV*_ CT attenuation on 100-keV virtual monochromatic images

### Prediction model evaluation

**Fig. 3:**
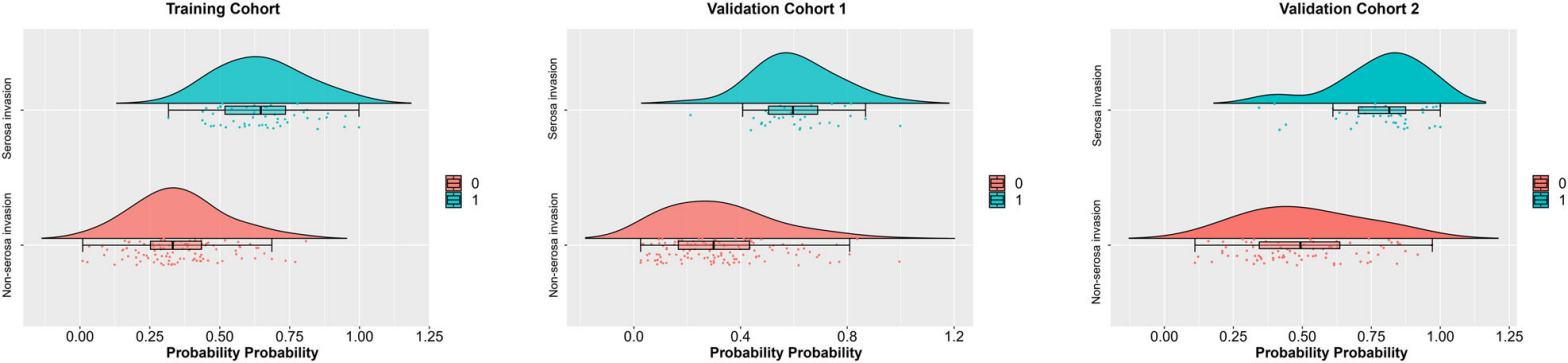
Raincloud plot of predicted probability between patients with and those without serosal invasion. It shows patterns of correlation between pathologic serosal status and DECT model in TC, VC1, and VC2, respectively. (0: without serosal invasion, 1: with serosal invasion)

**Fig. 4:**
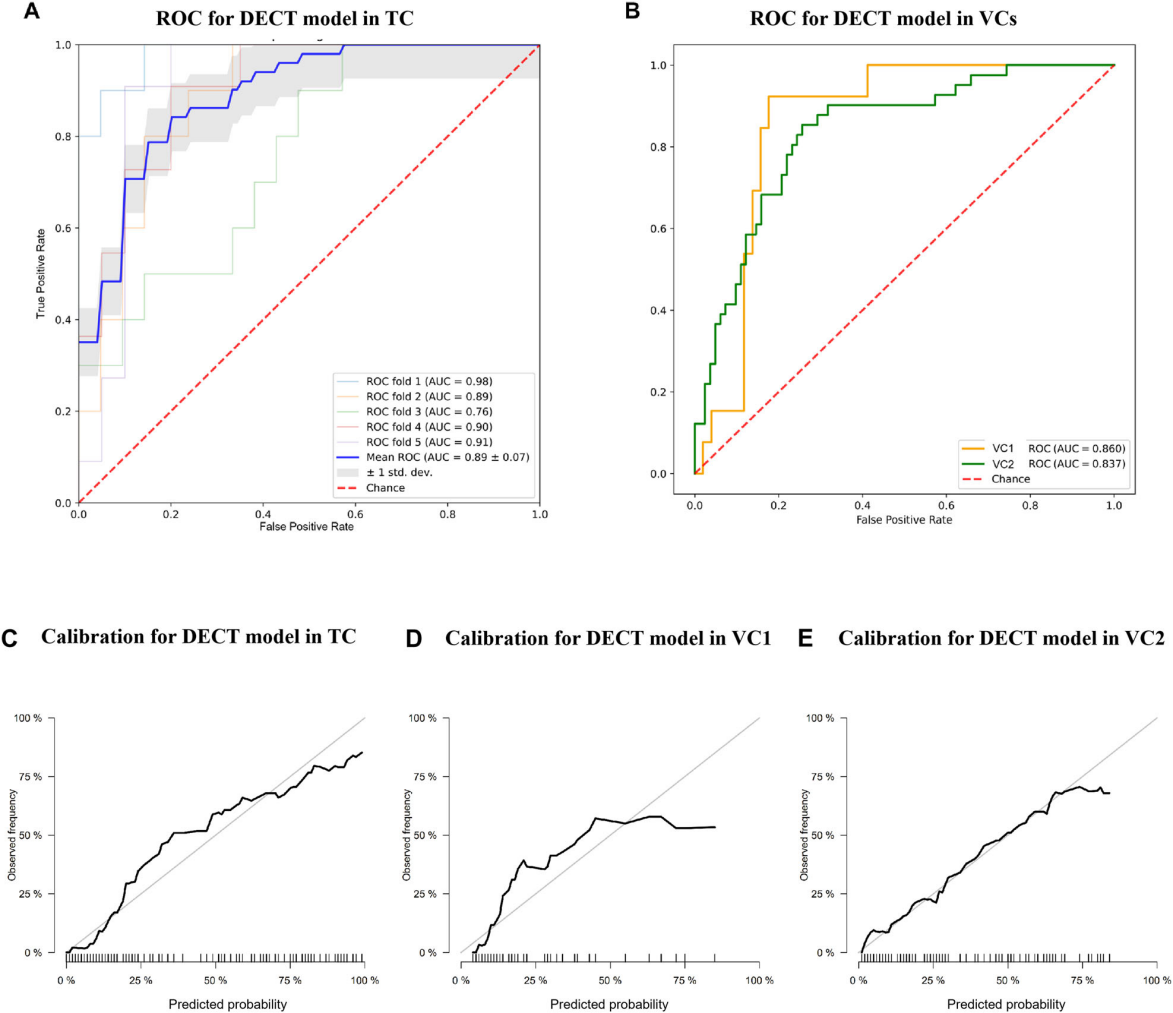
Assessment of DECT model for the discriminative ability and calibrations. Receiver operating characteristic (ROC) curve for the (**A**) training cohort, and (**B**) validation cohorts. Calibration curves of DECT model in all three cohorts (**C**, **D**, and **E**)

As shown in Fig. 3, the DECT model’s predicted probability was significantly different between the SI and the non-SI groups (Mann–Whitney *U*-test, all *p* < 0.0001). The model predicted SI with mean AUC value of 0.889 ± 0.07 in TC (detailed AUC results in the fivefold validation are shown in Fig. 4A). Good discriminative capability was also achieved in the VCs, with AUC values of 0.860 (95% CI: 0.775, 0.945) for VC1 and 0.837 (95% CI: 0.771, 0.902) for VC2 (Fig. 4B). Among the three cohorts, there were no significant differences in AUC values (Hanley–McNeil test: TC vs. VC1 *p* = 0.976; TC vs. VC2 *p* = 0.965; VC1 vs. VC2 *p* = 0.983). Regarding the prediction of serosal invasion, the AUC value of the DECT model in all cohorts was higher than that of any individual DECT quantitative parameters (Delong test, all *p* < 0.05); it demonstrated a significantly higher AUC in TC and VC2 (Delong test, both *p* < 0.05) compared to CT-reported T4a (Table 3 and Supplementary Table S3).

**Table 3:** Diagnostic performance of DECT model and predictors

Variable	TC						VC1				VC2			
	AUC	ACC	SEN	SPE	Threshold Value		AUC	ACC	SEN	SPE	AUC	ACC	SEN	SPE
DECT model	0.889	0.800	0.785	0.807	0.494^a^	-	0.860	0.813	0.615	0.863	0.837	0.650	0.902	0.524
	(0.825, 0.952)						(0.775, 0.945)				(0.771, .902)			
CT-reported T4a	0.679	0.645	0.787	0.571	-	-	0.728	0.703	0.769	0.686	0.515	0.520	0.512	0.524
	(0.598, 0.761)						(0.619, 0.837)				(0.418, 0.610)			
IC_VP	0.611	0.587	0.522	0.621	0.404^a^	18.06^b^	0.573	0.578	0.692	0.549	0.701	0.642	0.707	0.610
	(0.463, 0.759)						(0.452, 0.694)				(0.605, 0.793)			
VMI_70keV__VP	0.609	0.6	0.538	0.63	0.446^a^	81.87^b^	0.475	0.484	0.462	0.49	0.677	0.593	0.634	0.573
	(0.502, 0.717)						(0.353, 0.597)				(0.565, 0.770)			
VMI_100keV__VP	0.616	0.574	0.575	0.572	0.508^a^	57.98^b^	0.465	0.516	0.538	0.51	0.663	0.610	0.659	0.585
	(0.521, 0.711)						(0.342, 0.587)				(0.551, 0.759)			

*AP* arterial phase, *IC* iodine concentration, *VP* venous phase, *VMI*_*70keV*_ CT attenuation on 70-keV virtual monochromatic images, *VMI*_*100keV*_ CT attenuation on 100-keV virtual monochromatic images

^a^ The threshold of predicted probability

^b^ The threshold of raw value

According to the calibration curve, there was no significant deviation between the DECT model-predicted outcomes and the actual serosal status across all cohorts (Hosmer-Lemeshow test: TC, *p* = 0.120; VC1, *p* = 0.096; VC2, *p* = 0.865) (Fig. 4C–E). The stratification analysis demonstrated that our model performance was unaffected by age, tumor location, size (thickness), and differentiation degree (Hanley–McNeil test, all *p* > 0.05) (Supplementary Fig. S1).

**Fig. 5:**
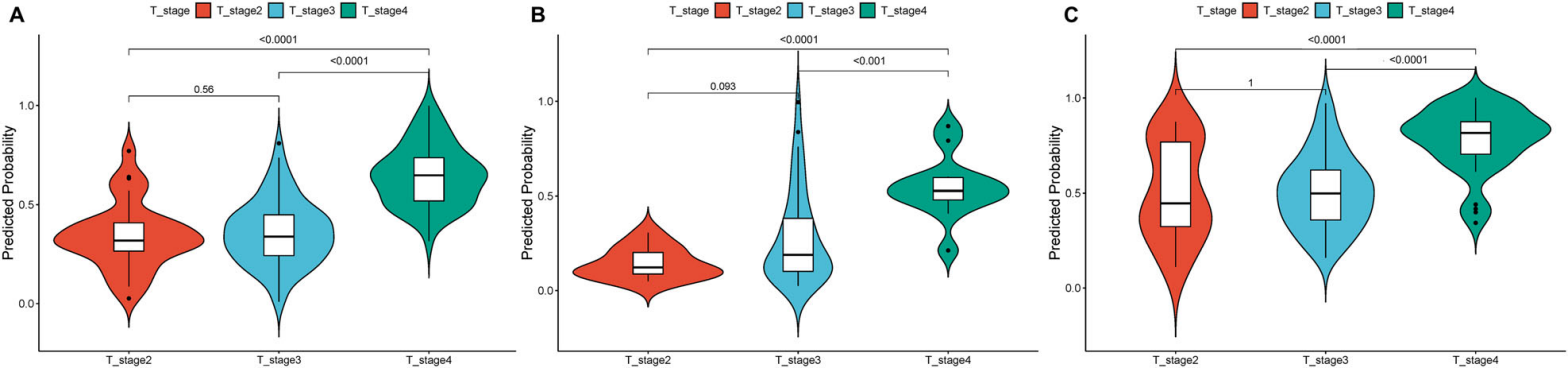
Subgroup analysis of the DECT model. Significant differences of predicted probability were observed between T3 and T4a and between T2 and T4a in the TC (**A**), VC1 (**B**), and VC2 (**C**)

As shown in Fig. 5, the DECT model could well discriminate T3 from T4a groups and T2 from T4a groups in all cohorts (Mann–Whitney *U*-test, all *p* < 0.001). Moreover, Spearman’s correlation coefficients confirmed the DECT model-predicted probability was significantly and positively correlated with pathologic T stages (0.476–0.610, *p* < 0.0001) (Supplementary Table S4). Importantly, our developed model could detect 57.1% (8/14) of patients with occult serosa invasion (CT-reported: SI-negative, postoperative pathology: SI-positive), suggesting its sensitivity to subtle changes in the serosa (Supplementary Fig. S2). If we assess the association of the DECT model with the N stage, as displayed in Supplementary Fig. S3, the beeswarm maps indicated that the model could only discriminate N0 from non-N0 groups in VC1 (*p* < 0.01).

### Follow-up and survival analysis

Among the 219 patients in TC and VC1, 213 patients were successfully followed up. Of these, 159 (74.6%; 159/213) received postoperative adjuvant therapy. The overall disease progression rate was 22% (47 of 213 patients), while the overall mortality rate was 10% (22 of 213 patients). The medians of DFS and OS for patients were 12.5 months and 14.8 months, respectively.

**Fig. 6:**
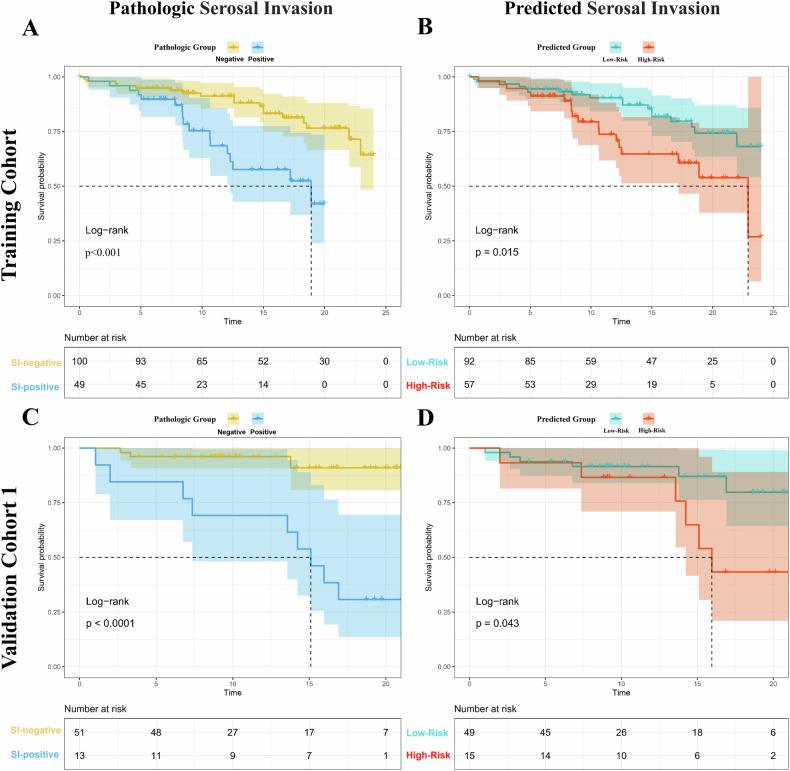
Kaplan–Meier survival curve of disease-free survival (DFS) stratified by the pathologic serosal invasion and the DECT model. Kaplan–Meier survival curve analysis shows the significant differences of DFS (**A**, **C**) between pathologic negative and positive serosal invasion groups (TC, *p* < 0.001; VC1, *p* < 0.0001) and (**B**, **D**) between predicted low- and high-risk serosal invasion groups (TC, *p* = 0.015; VC1, *p* = 0.043)

To assess the prognostic potential of the DECT model, patients were divided into high-risk (predicted probability ≥ 0.494) and low-risk (predicted probability < 0.494) groups. The optimal cutoff value of 0.494 was determined by the Youden index. In TC and VC1, our analyses showed significant differences in DFS between patients in the two risk groups (TC: hazard ratio of 2.233 [1.144–4.356], log-rank test, *p* = 0.015; VC1: hazard ratio of 3.042 [0.979–9.452], log-rank test, *p* = 0.043). On the other hand, pathologic SI was also found to be significantly associated with DFS in both cohorts (TC: hazard ratio of 3.081 [1.544–6.149], log-rank test, *p* < 0.001; VC1: hazard ratio of 9.479 [2.531–35.5], log-rank test, *p* < 0.0001) (Fig. 6). Supplementary Appendix S5 and Fig. S4 illustrates the correlation between the two and OS.

## Discussion

The aim of our study was to develop and validate a prediction model based on DECT to identify SI in patients with LAGC. We concluded that DECT parameters incorporated into easy-to-access LR classifiers could provide favorable performance. Specifically, in this multicenter study, the developed DECT model had good discriminative ability (AUC ranged from 0.837 to 0.889 for all three cohorts). Similar diagnostic performances of the DECT model were observed in the TC and two VCs (an independent external center and a cohort of other timelines), suggesting the robustness and generalizability of our model. As with pathologically confirmed SI, the DECT model was significantly associated with DFS in followed-up cohort.

In addition to suggesting potential micro-metastases and peritoneal spreading, serosal invasion is closely related to the choice of patient treatment options [20, 22, 23]. Neoadjuvant chemotherapy (NAC), used prior to LAGC surgery, is gaining popularity [24]. However, NAC is not currently the first option for LAGC in China. Also, this promising treatment scheme does not benefit all patients [25]. A PRODIGY study in Korea noted that neoadjuvant therapy benefits appeared greatest in patients with more advanced disease [26]. Hence, similar to artificial intelligence [27], our DECT model is also expected to aid the multidisciplinary team in making more personalized management decisions for LAGC patients. This involves deciding whether to conduct comprehensive and meticulous peritoneal exploration and whether to implement NAC. Of course, other factors, such as the patient’s physical condition, tumor location, size, and clinical lymph node stages, must also be considered in the decision-making process. Furthermore, as with previous studies [28, 29], we found that postoperative pathology-derived SI status was strongly associated with patients’ survival. Further analysis showed that the DECT model-predicted SI status also had prognostic value in predicting DFS, indicating that the developed model could potentially be an attractive surrogate for postoperative pathology-determined SI detection. The present study also validated the association between our model and the T stages; interestingly, it had the potential to discriminate T2 versus T4a, and T3 versus T4a in all three cohorts.

Although the conventional serosal invasion assessment criteria were used in this study [9], the diagnostic performance of CT-reported T4a was unsatisfactory with the AUC of 0.515–0.728. In 2020, You et al [30] reaved that SI could be diagnosed with 79.25–88.68% accuracy using qualitative radiological assessment. The differences in radiological qualitative assessment results between the two studies may stem from the large discrepancy in sample sizes (their vs our study, 53 vs 342 cases) and the use of different evaluation criteria for SI. Inspiringly, our proposed hybrid model demonstrated better predictive performance compared to single qualitative, morphological feature (CT-reported T4a) in TC and VC2 (Delong test: both *p* < 0.001).

Dual-phase enhancement has been the mainstream protocol for DECT in the GC field. The hemodynamics in the AP represents the capillary density and blood supply of the tumor, whereas VP imaging plays a role in reflecting the diffusion and retention of contrast agents in the interstitial space [15, 31, 32]. Although several DECT studies on GC highlight the advantages of AP parameters over VP parameters [13, 15], most research indicates that the latter has greater diagnostic value than the former [14, 16, 17, 32–34]. The VP-based DECT model that could provide individualized prediction of LNM preoperatively was first reported by Li et al [18] in 2018. Similarly, our study found that the independent predictors of SI, including IC, VMI_70keV_, and VMI_100keV_, were derived from the venous phase CT images. Thus, our study reaffirms that the VP is the ideal phase for GC imaging and that VP-based DECT is a useful tool for noninvasive TNM staging in GC [14, 18, 19, 35–37].

A prior DECT study has demonstrated that IC could detect SI in gastric cancer. Although they focused on IC in perigastric fat, both their results and ours show that cases of SI-positive have a higher IC value in VP [20]. Beyond just iodine parameters, there’s a growing exploration into the clinical utility of various other DECT parameters [16, 32, 34, 38]. Theoretically, energy-dependent attenuation changes in tissue offer abundant quantitative information [39], motivating the inclusion of VMIs at various energy levels in this study. Our findings demonstrate that attenuation values on both VMI_70keV_ and VMI_100keV_ were independent predictors in SI prediction. Previously, one study revealed that both 70 keV and 100 keV radiomics models based on VMI were superior to 40 keV models in predicting treatment response to systemic chemotherapy for advanced GC [40]. The similarity in these findings can be attributed to the fact that VMI_70keV_ and VMI_100keV_ are capable of better reflecting tumor characteristics as they strike a reasonable compromise between image noise and lesion clarity. In contrast, VMI_40keV_ and VMI_140keV_ are limited by significant image noise and lack of enhanced tissue characteristics, respectively [39, 41]. Notably, among the predictors used in the model, the VMI_70keV_ venous phase attenuation was negatively correlated with SI, whereas the VMI_100keV_ venous phase attenuation showed a positive correlation. Interestingly, during model construction, we found that excluding either of the two predictors did not alter the direction of the influence of the remaining predictor, but it did significantly decrease the model’s performance. Thus, there is no collinearity between the two. We speculate that these findings are due to the presence of adenocarcinoma types with different enhancement patterns in the SI-positive group. For example, some adenocarcinoma tumor cells can secrete a large amount of mucus and show less enhancement in most areas of the lesion on VP images (a lower attenuation at VMI_70keV__VP indicates a higher likelihood of SI) [42]; however, such enhancement pattern does not detract from the fact that it is highly invasive.

Our study has several limitations. First, to develop an individualized SI diagnostic tool that is easy to use and avoids ‘black boxes’, the DECT model was not built with complex machine learning algorithms. Second, due to the high rate of lost follow-up in VC2, no survival analysis was performed for this cohort. Third, all DECT images were acquired on rapid tube voltage-switching scanners (GE) at all centers; our results need further validation across different DECT platforms (e.g., dual-source system and dual-layer spectral detector system). Additionally, postoperative adjuvant therapy may affect DFS, highlighting the urgent need for a rigorously designed randomized controlled trial in the future.

In conclusion, the LR model from preoperative DECT has potential to serve as a noninvasive, quantitative tool for predicting serosal invasion in patients with LAGC. It can be used to stratify LAGC patients according to DFS. Additionally, this model, which is easy to obtain, could be valuable in preventing the oversight of occult peritoneal metastasis in individuals positive for serosal invasion. Further studies are required to test the generalizability of our model in more institutions and convert our findings into clinical practice.

## Supplementary information

ELECTRONIC SUPPLEMENTARY MATERIAL

## Data Availability

Data are available on reasonable request.

## Abbreviations

AJCC: American Joint Committee on Cancer
AP: Arterial phase
AUC: Area under the curve
CT: Computed tomography
DECT: Dual-energy CT
DFS: Disease-free survival
HER-2: Human epidermal growth factor receptor 2
IC: Iodine concentration
IM: Iodine maps
LAGC: Locally advanced gastric cancer
LNM: Lymph node metastasis
LR: Logistic regression
NAC: Neoadjuvant chemotherapy
nIC: Normalized IC
OS: Overall survival
ROC: Receiver operating characteristic
TC: Training cohort
TNM: Tumor, node, metastasis
VMI: Virtual monochromatic image
VP: Venous phase
WC: Water concentration
