# Supplementary material for: A quantitative model using multi-parameters in dual-energy CT to preoperatively predict serosal invasion in locally advanced gastric cancer
Source: Insights Imaging. 2024 Oct 31;15:264. doi: 10.1186/s13244-024-01844-z (PMC11528085; doi:10.1186/s13244-024-01844-z)
Supplement: Supplementary file 1 — ELECTRONIC SUPPLEMENTARY MATERIAL [file 13244_2024_1844_MOESM1_ESM.docx]

**Supplementary Material**

**Supplementary Appendix S1.** Patients with GC who underwent preoperative DECT and subsequent radical gastrectomy at six centers were consecutively enrolled: Center 1 (between November 2021 and May 2023; between December 2013 and December 2016), Center 2 (between August 2022 and June 2023), Center 3 (between August 2022 and June 2023), Center 4 (between September 2022 and June 2023), Center 5 (between February 2022 and June 2023), and Center 6 (between March 2022 and April 2023).

**Supplementary Appendix S2.**

Patients were prepared by fasting for 6-8 hours and drinking warm water (1000-1200 mL) before CT scanning in order to fully distend the stomach. They were asked to remain in the supine position, or in special cases, in the prone or left lateral position during the CT scan. The real position was adjusted according to the location of the lesion.

**Supplementary Appendix S3.**

An abdominal radiologist with 10 years of experience prospectively assessed and measured both qualitative and quantitative tumor information. The radiologist was informed that all patients had GC but were blinded to the clinical and postoperative pathological results. First, the following morphological features of tumors for each patient were assessed: (a) tumor location (upper vs middle vs lower vs diffuse), (b) tumor thickness, (c) CT-reported T4a (negative vs positive): positive was defined by the serosal/outer surface displays an irregular or nodular morphology, with adjacent adipose space exhibiting dense spicular or band-like infiltration, (d) CT-reported LNM (negative vs positive): positive was defined by a short diameter of the lymph node greater than 8 mm, significant or heterogeneous enhancement, or multiple clusters of lymph nodes (three or more)[1]. Second, iodine concentration (IC), water concentration (WC), and monochromatic attenuation were measured on IM, WM, and VMIs of various energy levels, respectively, by delineating the region of interest (ROI) at the largest circumference of the tumor and its adjacent upper and lower slices. The ROI covered approximately two-thirds of the lesion area and excluded obvious calcification, surrounding adipose tissue, and vessels. Additionally, the abdominal aortic IC values were obtained by placing circular ROI on the same slices. Next, the normalized IC (nIC), which is the ratio of the tumor IC to the aortic IC (nIC=IC tumor/IC aorta), was calculated. The mean values for each parameter were obtained by averaging the measurements from the ROIs of the three consecutive image slices. All morphological features and depiction of ROI were reviewed and confirmed by another senior radiologist (with 20 years of experience in abdominal radiologist).

**Supplementary** **Appendix S4.**

Selection process of independent predictors: First, we examined correlations between the 14 DECT quantitative parameters via the spearman correlation analysis. If the correlation coefficient between two features is greater than 0.9, the one of the two that is also highly correlated with the other parameters will be eliminated. Second, univariable analysis was performed to select preoperative baseline variables associated with serosal invasion among age, sex, tumor differentiation degree, 4 morphological features, and all non-redundancy DECT parameters. Significant factors (p<0.05) at univariable analysis were included in the multivariate logistic regression. A stepwise regression procedure was then employed to identify independent predictors for serosal invasion.

**Supplementary Appendix S5.**

Since the follow-up time was short, there was no statistical prognostic significance observed in pathologic serosal invasion for OS in the TC (Log-rank, P=0.86), or in the DECT model for OS in the TC (Log-rank, P=0.76) and VC1 (Log-rank, P=0.72). Only the patients with positive serosal invasion had shorter OS in the VC1 than those negative serosal invasion (hazard ratio of 6.811 [1.23-37.72], log-rank, P=0.011) (Supplementary FigureS4).

**Supplementary Table S1**. The CT image acquisition parameters of the six centers

|  | **Center 1** | | **Center 2** | **Center 3** | **Center 4** | **Center 5** | **Center 6** |
| --- | --- | --- | --- | --- | --- | --- | --- |
| Period | 2021.11-2023.5 | 2013.12-2016.12 | 2022.8-2023.6 | 2022.8-2023.6 | 2022.9-2023.6 | 2022.2-2023.6 | 2022.3-2023.4 |
| CT scanner | Discovery GSI,  Revolution Apex | Discovery HD750 | Revolution | Revolution | Revolution | Revolution | Revolution |
| Scanning model | GSI | GSI | GSI | GSI | GSI | GSI | GSI |
| Tube voltage | between 80 and 140 kVp | | between 80 and 140kVp | between 80 and 140kVp | between 80 and 140kVp | between 80 and 140kVp | between 80 and 140kVp |
| Pitch | 1.375 | | 1.375 | 1.375 | 1.375 | 1.375 | 0.992 |
| Scan field of view | Large Body | | Large Body | Large Body | Large Body | Large Body | Large Body |
| Rotation time | 0.6 s | | 0.8 s | 0.8 s | 0.8 s | 0.8 s | 0.8 s |
| Detector width | 40mm，80mm | 40mm | 80mm | 80mm | 80mm | 80mm | 80mm |
| Tube current | 375mA，405mA | 375mA | 400mA | 400mA | 400mA | 400mA | 400mA |
| Contrast agent |  |  |  |  |  |  |  |
| *Concentration* | 350mgI/ml | 350 or 370 mgI/ml | 350mgI/ml | 350mgI/ml | 350mgI/ml | 350mgI/ml | 350mgI/ml |
| *Dosage* | 1.2-1.3 ml/kg | 1.5ml/kg | 1.2-1.3 ml/kg | 1.2-1.3ml/kg | 1.2-1.3 ml/kg | 1.2-1.3ml/kg | 1.2-1.3ml/kg |
| *Infused rate* | 2-4 mL/s | 2-4 mL/s | 2-4 mL/s | 2-4 mL/s | 2-4 mL/s | 2-4 mL/s | 2-4 mL/s |
| Arterial phase CT | 11s after triggering the intra-abdominal aortic threshold (100 HU) | 30s after injection | 11s after triggering the intra-abdominal aortic threshold (100 HU) | 11s after triggering the intra-abdominal aortic threshold (100 HU) | 11s after triggering the intra-abdominal aortic threshold (100 HU) | 11s after triggering the intra-abdominal aortic threshold (100 HU) | 5.4s after triggering the intra-abdominal aortic threshold (110 HU) |
| Venous phase CT | 30s after completing the AP acquisition | 70s after injection | 30s after completing the AP acquisition | 30s after completing the AP acquisition | 30s after completing the AP acquisition | 30s after completing the AP acquisition | 25s after completing the AP acquisition |
| Image matrix | 512×512 | | 512×512 | 512×512 | 512×512 | 512×512 | 512×512 |
| Reconstruction section increment | 1.25 mm | | 1.25 mm | 1.25 mm | 1.25 mm | 1.25 mm | 1.25 mm |
| Reconstruction section thickness | 1.25 mm | | 1.25 mm | 1.25 mm | 1.25 mm | 1.25 mm | 1.25 mm |

GSI, gemstone spectral imaging

**Supplementary TableS2. Clinicopathological characteristics in the training and validation cohorts**

| **Variable** | Training cohort  (n=155) | Validation cohort1  (n=64) | Validation cohort2  (n=123) | P value |
| --- | --- | --- | --- | --- |
| **Age** | 62.910±9.836 | 63.078±8.512 | 59.122±10.949 | 0.003 ** |
| **Thickness**  **(mm)** | 17.466±6.821 | 15.942±6.486 | 16.494±6.620 | 0.243 |
| **Sex** |  |  |  | 0.951 |
| female | 31 (20.0%) | 13 (20.3%) | 23 (18.7%) |  |
| male | 124 (80.0%) | 51 (79.7%) | 100 (81.3%) |  |
| **Location** |  |  |  | 0.156 |
| upper | 58 (37.4%) | 31 (48.4%) | 44 (35.8%) |  |
| middle | 25 (16.1%) | 9 (14.1%) | 19 (15.4%) |  |
| lower | 57 (36.8%) | 13 (20.3%) | 48 (39.0%) |  |
| diffuse^a^ | 15 (9.7%) | 11 (17.2%) | 12 (9.8%) |  |
| **LNM** |  |  |  | 0.052 |
| negative | 36 (23.2%) | 19 (29.7%) | 45 (36.6%) |  |
| positive | 119 (76.8%) | 45 (70.3%) | 78 (63.4%) |  |
| **Differentiation degree** |  |  |  | 0.056 |
| non-poorly  differentiated | 95 (61.3%) | 28 (43.8%) | 71 (57.7%) |  |
| poorly  differentiated | 60 (38.7%) | 36 (56.2%) | 52 (42.3%) |  |

Note: ^a^ Diffuse ≥ 2/3 stomach.

LNM, Lymph node metastasis.

The comparison of Age, and Thickness between the three groups using the Analysis of variance;

The comparison of Sex, CT-reported T4a, CT-reported LNM, Location, LNM, and Differentiation degree between the three groups using the chi-squared test.

**Supplementary Table S3.1 Comparison of discrimination of DECT model and predictors in TC**

| **CT reported T4a** | <0.0001 |  |  |  |
| --- | --- | --- | --- | --- |
| **IC_VP** | <0.0001 | 0.293 |  |  |
| **VMI_70keV__VP** | <0.0001 | 0.301 | 0.928 |  |
| **VMI_100keV__VP** | <0.0001 | 0.339 | 0.859 | 0.803 |
|  | **DECT model** | **CT reported T4a** | **IC_VP** | **VMI_70keV__VP** |

AP, arterial phase; IC, iodine concentration; VP, venous phase; VMI, virtual monochromatic images.

VMI_70keV_, CT attenuation on 70-keV virtual monochromatic images; VMI_100keV_, CT attenuation on 100-keV virtual monochromatic images.

**Supplementary Table S3.2 Comparison of discrimination of DECT model and predictors in VC1**

| **CT reported T4a** | 0.080 |  |  |  |
| --- | --- | --- | --- | --- |
| **IC_VP** | <0.0001 | 0.145 |  |  |
| **VMI_70keV__VP** | <0.0001 | 0.034 | 0.065 |  |
| **VMI_100keV__VP** | <0.0001 | 0.026 | 0.195 | 0.797 |
|  | **DECT model** | **CT reported T4a** | **IC_VP** | **VMI_70keV__VP** |

AP, arterial phase; IC, iodine concentration; VP, venous phase; VMI, virtual monochromatic images.

VMI_70keV_, CT attenuation on 70-keV virtual monochromatic images; VMI_100keV_, CT attenuation on 100-keV virtual monochromatic images.

**Supplementary Table S3.3 Comparison of discrimination of DECT model and predictors in VC2**

| **CT reported T4a** | <0.0001 |  |  |  |
| --- | --- | --- | --- | --- |
| **IC_VP** | 0.001 | 0.017 |  |  |
| **VMI_70keV__VP** | 0.001 | 0.041 | 0.455 |  |
| **VMI_100keV__VP** | <0.001 | 0.056 | 0.431 | 0.527 |
|  | **DECT model** | **CT reported_T4** | **IC_VP** | **VMI_70keV__VP** |

AP, arterial phase; IC, iodine concentration; VP, venous phase; VMI, virtual monochromatic images.

VMI_70keV_, CT attenuation on 70-keV virtual monochromatic images; VMI_100keV_, CT attenuation on 100-keV virtual monochromatic images.

.

**Supplementary Table S4**. **DECT model evaluation: Subgroup analysis of pathologic T stages.**

|  | Training cohort | Validation cohort1 | Validation cohort2 |
| --- | --- | --- | --- |
| Spearman correlation coefficient | 0.610 (0.501-0.701) | 0.529(0.325-0.686) | 0.476 (0.326-0.602) |
| P value | 3.40×10^-17^ | 7.03×10^-6^ | 2.71×10^-8^ |


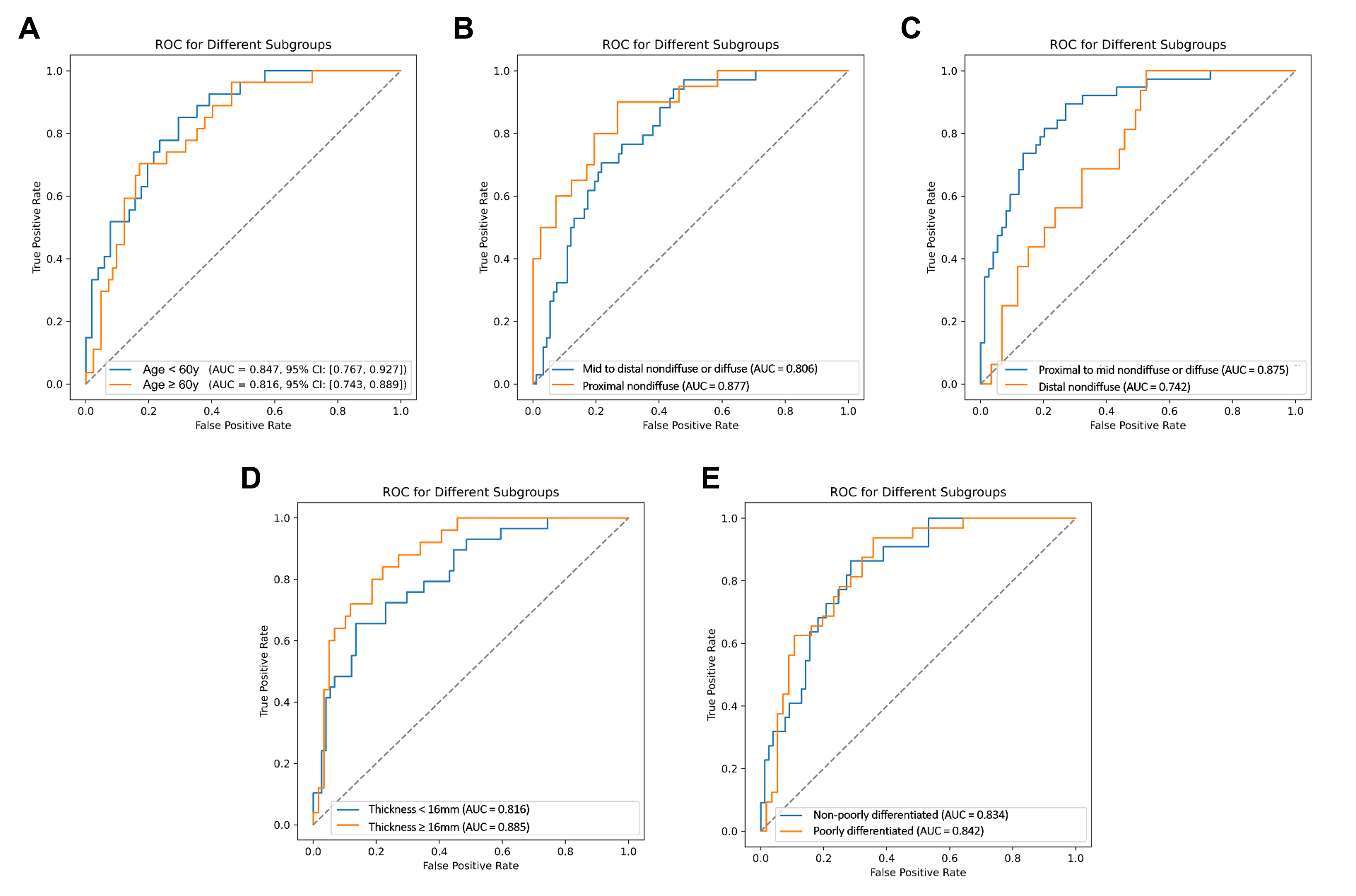


**Supplementary Figure S1. Stratification analysis of the DECT model in the combination of two validation data set (N=187).** Subgroup analysis were conducted on age (A), tumor location (B, C), thickness (D), and differentiation degree (E).


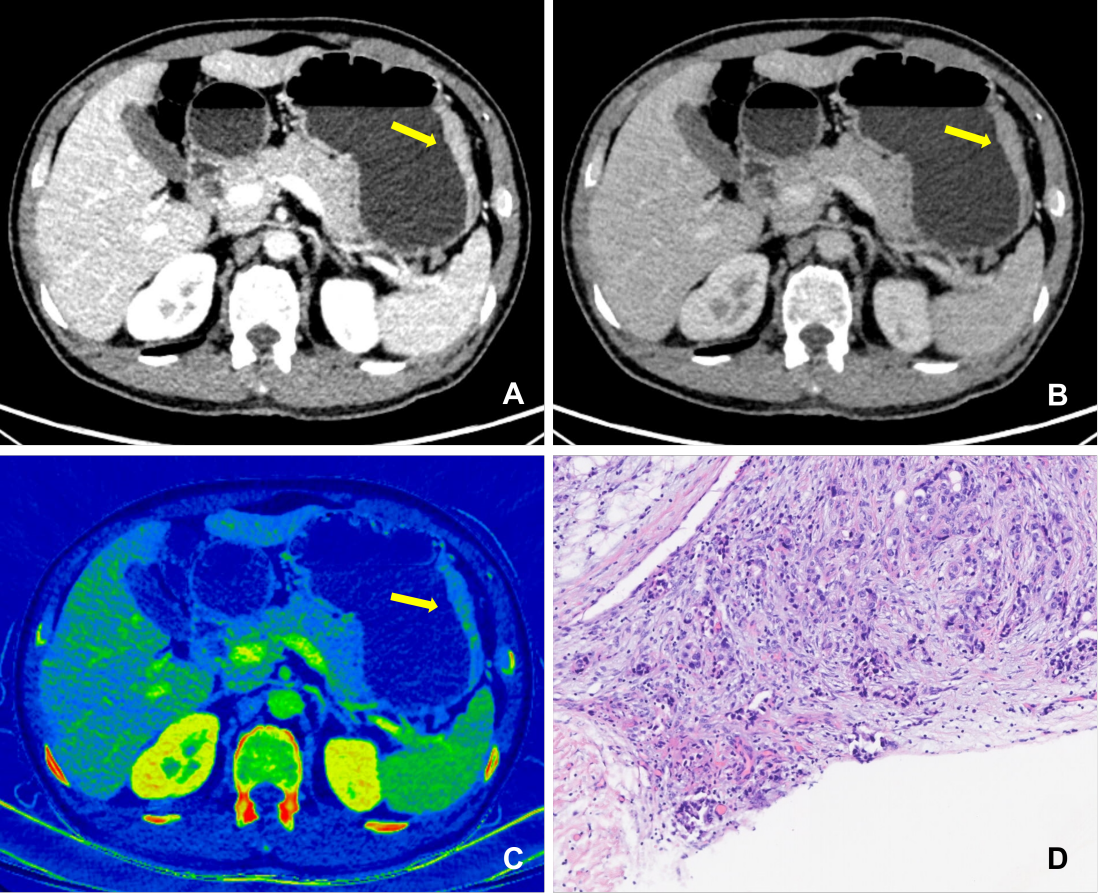


**Supplementary Figure S2. Venous phase-based DECT images and histopathology images in a case of gastric cancer with occult** **serosal invasion**. Male, 52 years old, pathologically confirmed gastric adenocarcinoma in the greater curvature of the gastric body, differentiated degree of II. The tumor presented localized thicken of the stomach wall with IC value, monochromatic attenuation values being 98.03 (HU), 59.96 (HU), and 25.94 (100ug/ml) measured on VMI_70keV_ (**A**), VMI_100keV_ (**B**), and iodine map (**C**), respectively. This case was regarded as serosal invasion-negative on CT, which are without typical CT signs of serosal invasion(i.e. a dense band-like fat infiltration around the stomach). However, this patient was assessed as serosal invasion-positive based on our DECT model, which is consistent with the finding of histopathology (**D**).


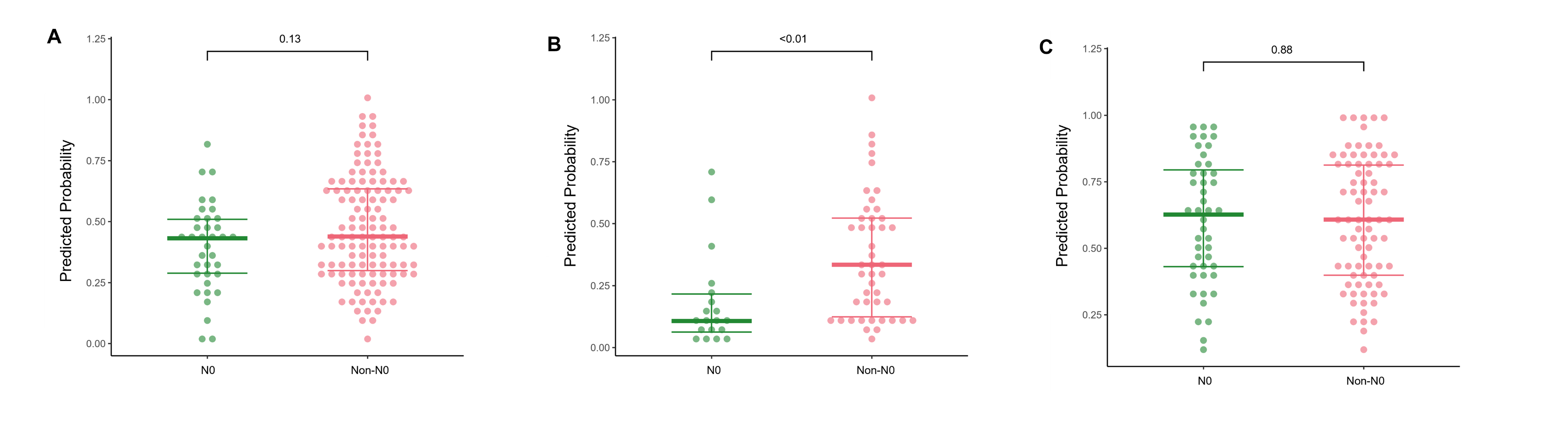


**Supplementary Figure S3.** Beeswarm maps of patients’ DECT model-predicted probability between N0 stage and non-N0 stage groups in training cohort (A), validation cohort 1 (B), and validation cohort 2 (C).


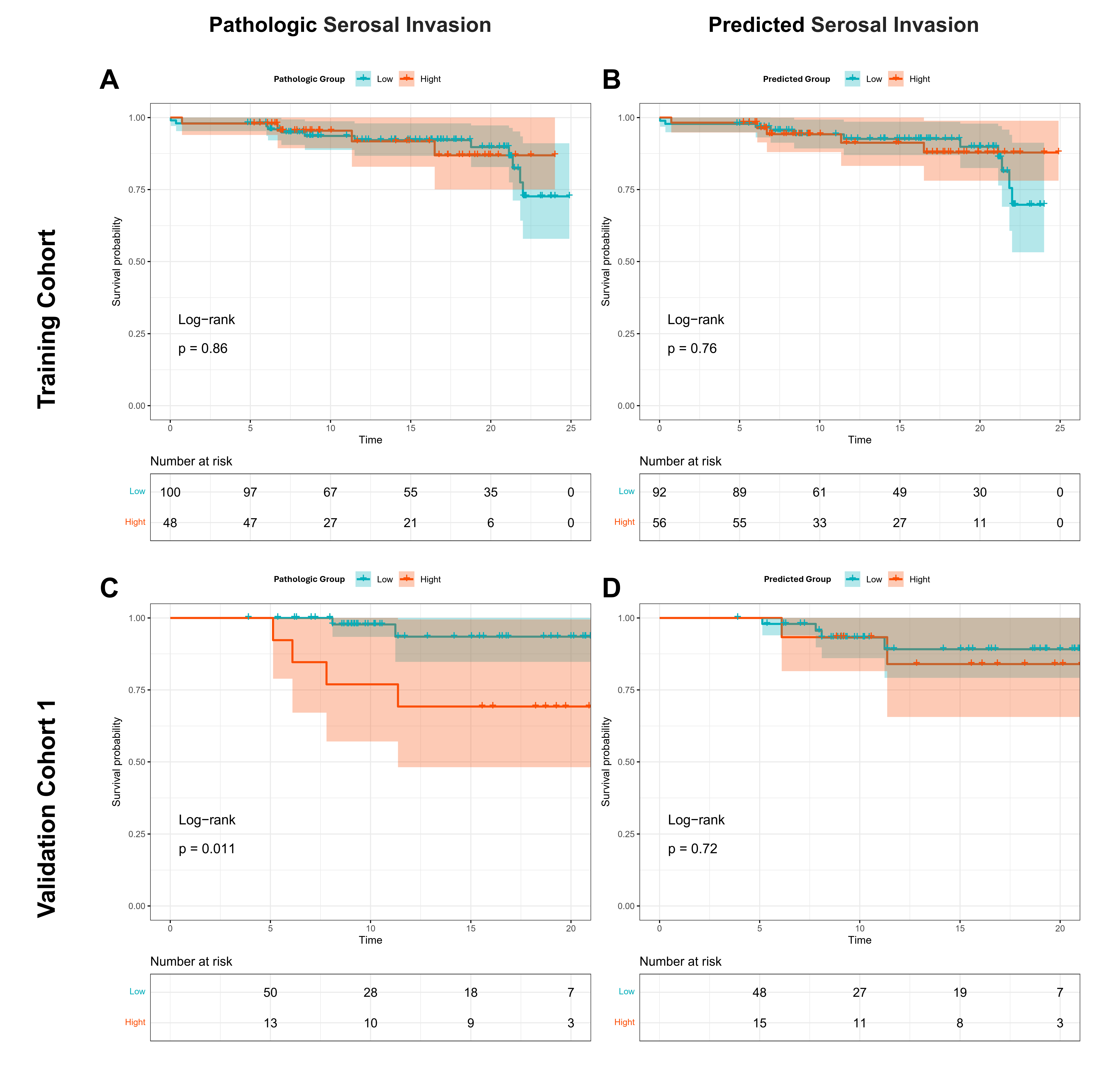


**Supplementary Figure S4.** Kaplan–Meier survival curve of overall survival (OS) stratified by the pathologic serosal invasion (A, training cohort; C,validation cohort 1) and the DECT model (B, training cohort; D,validation cohort 1).

1 Wang F-H, Zhang X-T, Tang L et al (2024) The Chinese Society of Clinical Oncology (CSCO): Clinical guidelines f or the diagnosis and treatment of gastric cancer, 2023. Cancer communications (London, England) 44:127-172
